# Investigating and Engineering an 1,2-Propanediol-Responsive Transcription Factor-Based Biosensor

**DOI:** 10.1021/acssynbio.4c00237

**Published:** 2024-07-05

**Authors:** Yuxi Teng, Xinyu Gong, Jianli Zhang, Ziad Obideen, Yajun Yan

**Affiliations:** † School of Chemical, Materials and Biomedical Engineering, College of Engineering, The University of Georgia, Athens, Georgia 30602, United States; ‡ Franklin College of Arts and Sciences, The University of Georgia, Athens, Georgia 30602, United States

**Keywords:** PocR, biosensor, 1,2-propanediol, short chain-alcohols, RNA polymerase recruiter, bifunctional regulation

## Abstract

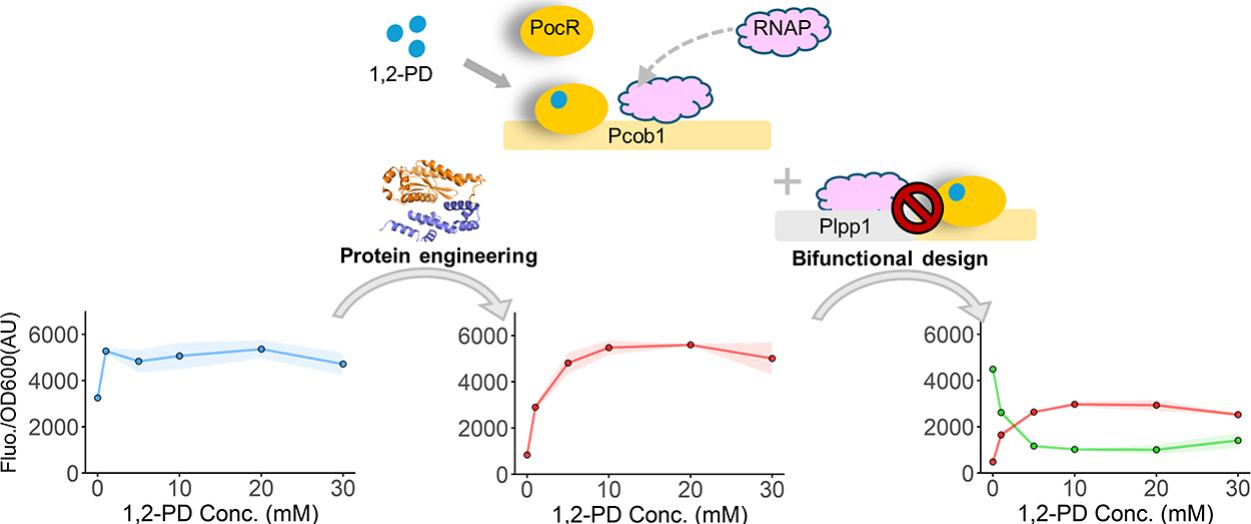

Transcription factor (TF)-based biosensors have arisen as powerful tools in the advancement of metabolic engineering. However, with the emergence of numerous bioproduction targets, the variety of applicable TF-based biosensors remains severely limited. In this study, we investigated and engineered an 1,2-propanediol (1,2-PD)-responsive transcription activator, PocR, from *Salmonella typhimurium* to enrich the current biosensor repertoire. Heterologous characterization of PocR in *E. coli* revealed a significantly limited operational range and dynamic range, primarily attributed to the leaky binding between PocR and its corresponding promoters in the absence of the 1,2-PD inducer. Promiscuity characterization uncovered the minor responsiveness of PocR toward glycerol and 1,2-butanediol (1,2-BD). Using AlphaFold-predicted structure and protein mutagenesis, we preliminarily explored the underlying mechanism of PocR. Based on the investigated mechanism, we engineered a PcoR-F46R/G105D variant with an altered inducer specificity to glycerol, as well as a PocR-ARE (Q107A/S192R/A203E) variant with nearly a 4-fold higher dynamic range (6.7-fold activation) and a 20-fold wider operational range (0–20 mM 1,2-PD). Finally, we successfully converted PocR to a repressor through promoter engineering. Integrating the activation and repression functions established a versatile 1,2-PD-induced bifunctional regulation system based on PocR-ARE. Our work showcases the exploration and exploitation of an underexplored type of transcriptional activator capable of recruiting RNA polymerase. It also expands the biosensor toolbox by providing a 1,2-PD-responsive bifunctional regulator and glycerol-responsive activator.

## 1 Introduction

Metabolic engineering strategically manipulates and reconstructs metabolic pathways in microbes to environmentally and sustainably produce a diverse array of compounds vital for industrial applications, such as fuels and pharmaceuticals.^1,2^ Despite significant advancements, there are still challenges in achieving optimal titers, yields, and productivities, including the difficulty to obtain effective genes and enzymes, as well as the imbalances and burdens brought by heterologous gene expression.^3−5^ Over the years, transcription factor (TF)-based biosensors have been playing a pivotal role in addressing these challenges.^6−8^ These biosensors can modulate gene expression in response to specific biomolecule signals. With fluorescence as output, TF-based biosensors have enabled the high-throughput screening of various overproducers for the biosynthesis of lactam,^9^l-cysteine,^10^*p*-coumaric acid,^11^ and butanol.^12^ In addition, biosensor-based dynamic regulation has efficiently increased the production of valuable compounds such as glucaric acid,^13^ rapamycin,^14^ 4-hydroxycoumarin,^15^ naringenin.^16^

However, the current biosensor toolbox remains highly limited, being capable of detecting only a small number of biomolecules. For example, the bioproduction of short-chain alcohols is of great interest due to their extensive applications in manufacturing pharmaceuticals, cosmetics, antifreeze, biofuel, and various polymers.^17−20^ To our knowledge, there are only two applicable biosensors for short-chain alcohols, including a butanol-responsive BmoR and an engineered isopentanol-responsive AlkS.^12,21^ Previous studies have reported an AraC family transcription factor PocR able to sense 1,2-propanediol (1,2-PD), which structurally distinguishes from butanol and isopentanol by the presence of two hydroxy groups, suggesting potentials for the relevant biosensing repertoire expansion.^22−24^ Nevertheless, PocR has not been explored for its potential in synthetic biology and metabolic engineering.

In this study, we introduced PocR and its corresponding promoters (Pcob and Ppdu) into *E. coli* and utilized eGFP as a reporter to study this regulation system. Initially, both Pcob and Ppdu showed low expression levels and required binding with PocR to efficiently recruit the RNA polymerase (RNAP). While PocR could function independently as an activator, adding its inducer 1,2-PD further enhanced the activation and triggered full expression. We also characterized the promiscuity of PocR with short chain alcohols sharing structures similar to those of 1,2-PD and observed the minor responsiveness to glycerol and 1,2-butanediol. Assisted by the AlphaFold-predicted protein structure, we investigated the underlying operational mechanism of PocR. Subsequent rational engineering generated a PocR variant (R46R/G106D) with altered inducer specificity to glycerol and an optimized variant PocR-ARE (Q107A/S192R/A203E) with an efficiently enhanced dynamic range and operational range. Lastly, we demonstrated the feasibility of converting PocR to a repressor on the specially designed promoters. This repression module can be combined with the activation module to develop a 1,2-PD-induced bifunctional regulation system with versatile performances. These engineered PocR variants and regulation circuits are suitable for various metabolic engineering applications. Moreover, our research highlights the substantial advantage of RNAP-recruiting biosensors such as PocR in expanding the functionality of synthetic circuits.

## 2 Results

### 2.1 Establishing and Characterizing the PocR Regulation System

**Figure 1:**
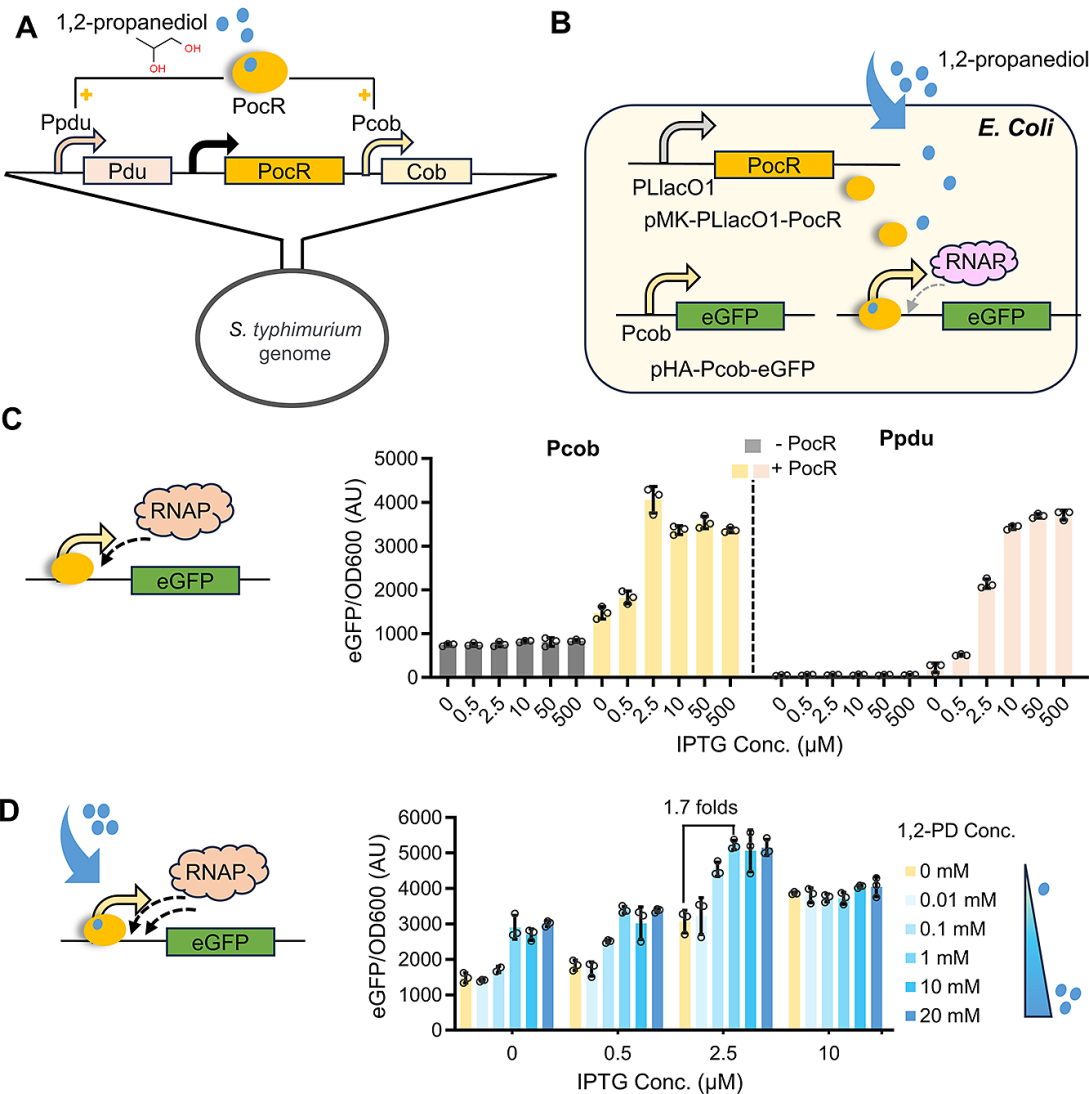
PocR regulation system development and characterization. (A) Illustration of PocR regulon in *S. typhimurium*. (B) Establishment of PocR regulation system in *E. coli*. (C) Fluorescence expression increased with increasing PocR levels by higher IPTG concentrations. (D) Fluorescence expression at increasing concentrations of the 1,2-PD effector. All the tests were performed with three independent biological repeats. Individual data points are represented by circles (○), and error bars indicate standard deviations (SD).

In *S. typhimurium*, PocR regulates the Cob operon (for vitamin B12 biosynthesis) and Pdu operon (for 1,2-PD degradation) by interacting with their respective promoters, Pcob and Ppdu (Figure 1A). To establish the PocR regulation system in *E. coli*, we inserted PocR downstream of the PLlacO1 promoter in the medium-copy number plasmid pMK-MCS. The corresponding promoter Pcob (or Ppdu) was used to control the expression of an eGFP reporter gene in the high-copy plasmid pHA-MCS (Figure 1B).

With the established regulation system, we observed only minimal expression of around 750 and 50 AU from Pcob and Ppdu in the absence of PocR, indicating the severely limited ability of these two promoters to harness RNAP (Figure 1C). The presence of PocR could vastly improve the expression from both promoters, with the maximal expression level reaching around 3500 AU. At certain PocR levels, adding the effector 1,2-PD could further induce stronger eGFP expression (Figures 1D and S1). Notably, when PocR was expressed by 2.5 μM IPTG, adding 1 mM 1,2-PD induced the highest fluorescence level of 5226 AU from the Pcob promoter, representing an operational range of 0–1 mM 1,2-PD and a dynamic range of 1.7-fold. As PocR was increasingly expressed by 10 μM IPTG, 1,2-PD addition barely caused any further activation. Similar performances, including almost the same operational range and dynamic range, were observed with the Ppdu promoter (Figure S1). These results validated that PocR can detect 1,2-PD and bind with its corresponding promoter to activate gene expression as a response. However, it is noticeable that PocR can independently activate gene expression without the presence of 1,2-PD. This leaky gene expression before inducer addition potentially causes a highly limited dynamic range and also explains the ineffective activation by 1,2-PD when PocR is expressed at excessive levels (Figures 1D and S1). While all the performances are consistent with previous *in vitro* characterizations,^22^ the narrow operational range and low dynamic range of PocR significantly impair its applicability and therefore require optimization.

**Figure 2:**
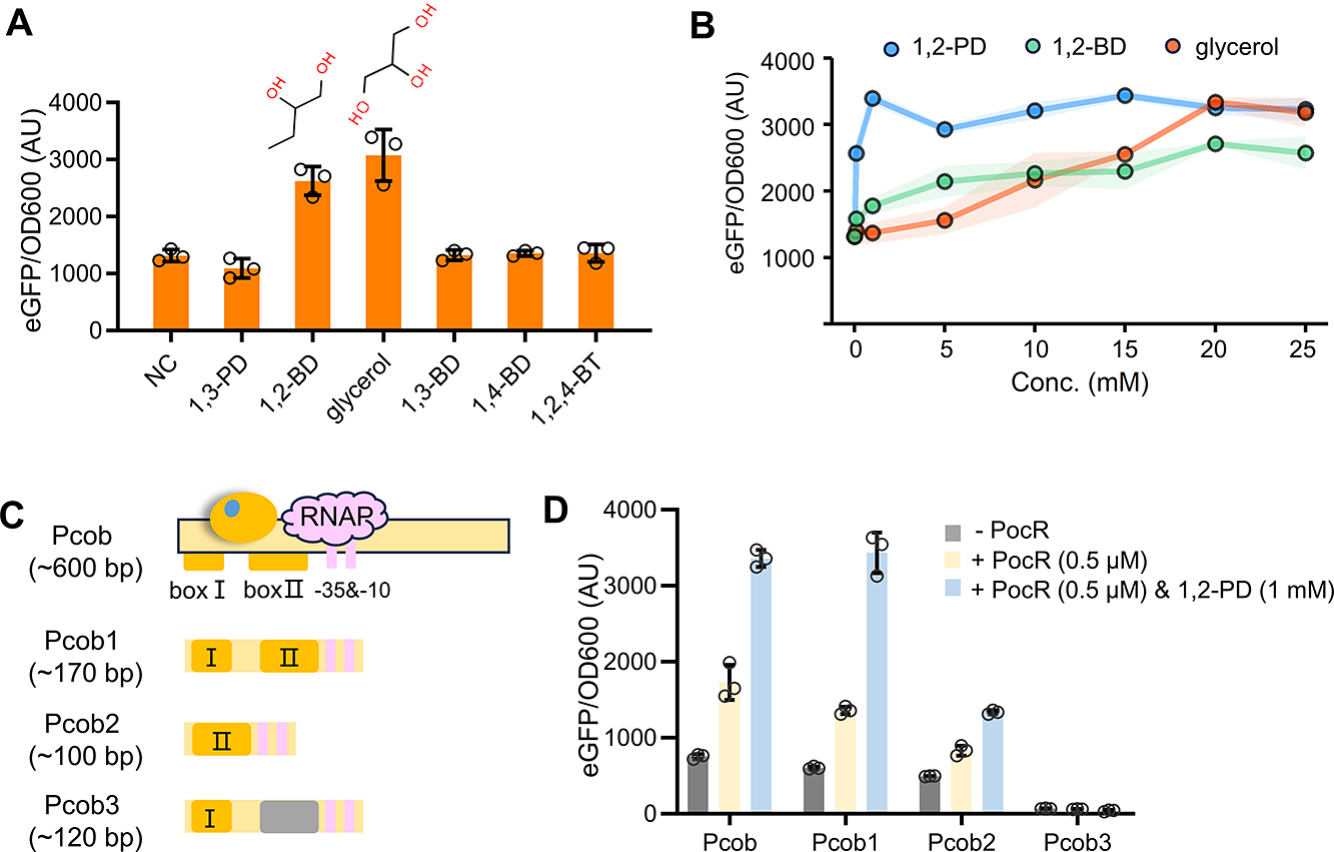
PocR promiscuity characterization and Pcob truncation. (A) Responsiveness of PocR to various short-chain alcohols. PocR was expressed by 0.5 μM IPTG, and all alcohols were added at a concentration of 20 mM. Individual data points are represented by circles (○), and error bars indicate the SD. (B) Comparison of different PocR effectors. The shaded regions represent standard deviation errors. PocR was consistently expressed with 0.5 μM IPTG. Shaded areas indicate the SD. (C) Schematic representation of truncated promoters. (D) Characterization of the truncated promoters. Individual data points are represented by circles (○), and error bars indicate the SD. All the tests were performed with three independent biological repeats.

In addition to 1,2-PD, we evaluated the promiscuity of PocR toward other short-chain alcohols with similar structures, including 1,3-propanediol (1,3-PD), 1,2-butanediol (1,2-BD), glycerol, 1,3-butanediol (1,3-BD), 1,4-butanediol (1,4-BD), and 1,2,4-butanetriol (1,2,4-BT) (Figure 2A). Results indicated that 1,2-BD and glycerol could also serve as effectors for PocR, although further comparison uncovered that PocR is most sensitive to the original inducer 1,2-PD (Figure 2B). With PocR expressed by 0.5 mM IPTG, 1 mM 1,2-PD can trigger full activation from 1420 to 3396 AU. Comparatively, PocR showed less responsiveness to glycerol or 1,2-BD at the same concentration. It required 20 mM of glycerol to trigger a full activation to 3335 AU, and 20 mM 1,2-BD could only induce the highest activation of 2709 AU. These results confirmed that PocR can detect not only 1,2-PD but also glycerol and 1,2-BD, though its activity is comparatively lower with the latter two inducers.

Next, we endeavored to explore and simplify the Pcob promoter. Previous *in vivo* analysis has identified two binding boxes within the Pcob promoter.^22^ Within the 600-bp Pcob regulatory region, the two identified binding boxes are 20 and 48 bp in length, respectively, distant by a 52-bp sequence (Figure 2C). Both boxes are located upstream of the −35 region, which is only 9 bp from binding box II. Based on that, we designed serial truncations of Pcob as shown in Figure 2C. Specifically, Pcob1 was truncated to only ∼170 bp, with all sequences after the theoretical transcription starting site removed. As a result, Pcob1 exhibited no noticeable differences compared to the wild type Pcob, demonstrating nearly the same activation levels by PocR and the same enhancement upon the addition of 1,2-PD (Figure 2D). We further removed binding box I of Pcob1 to generate Pcob2, which exhibited a significant loss of function. Pocb3 was generated using a random sequence to replace the binding box II in Pocb1. The absence of box II rendered Pcob3 completely unable to interact with PocR as well. These findings confirmed that binding boxes I and II are indispensable for the efficient interaction between the promoter and PocR. Furthermore, we ruled out the presence of any uncharacterized functional elements and obtained Pcob1 as a more streamlined promoter to be used in subsequent studies.

### 2.2 Predicting and Dissecting the Protein Structure of PocR

**Figure 3:**
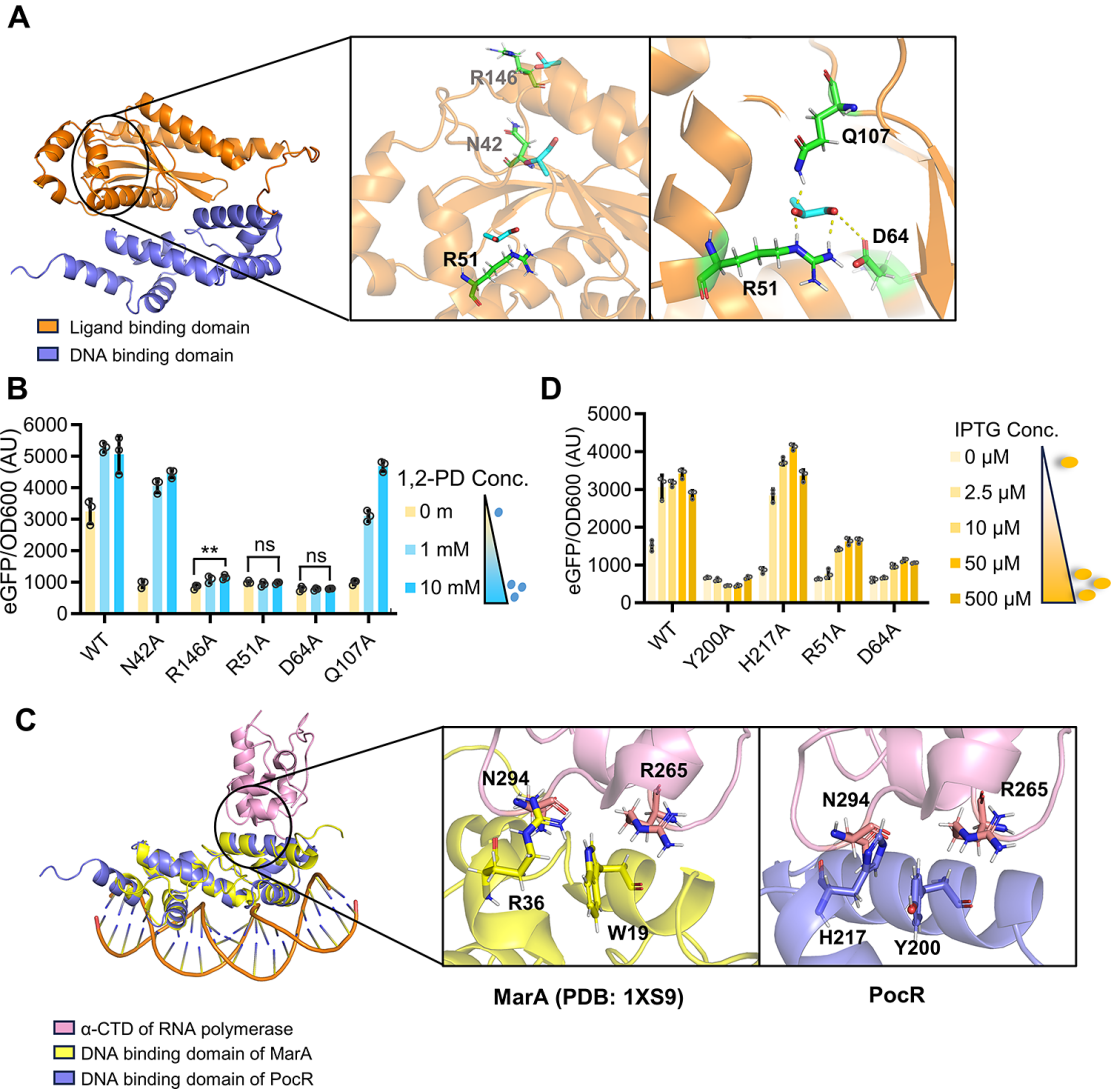
Prediction and investigation into the PocR regulation mechanism. (A) Schematic of the predicted PocR structure and zoom-in views of its potential ligand-binding residues. (B) Characterization of the responsiveness of different PocR variants to 1,2-PD. PocR was consistently expressed with 2.5 μM IPTG. Statistical analysis was performed using a two-tailed *t* test. ns, no significance; **, *P* < 0.01. (C) Structural overlay of the predicted DNA binding domain of PocR with the crystallized MarA:DNA:RNAP complex (PDB: 1XS9). Zoom-in views illustrate the interaction between the two regulators and RNAP. (D) Characterization of the activity of different PocR variants. All the tests were performed with three independent biological repeats. Individual data points are represented by circles (○), and error bars indicate SD.

To investigate the functional mechanism of PocR, we employed AlphaFold to predict its protein structure.^25^ As depicted in Figure 3A, PocR consists of an N-terminal ligand-binding domain (LBD, colored in orange) and a C-terminal helix-turn-helix DNA-binding domain (DBD, colored in purple).

The LBD of PocR is responsible for its interaction with the inducer 1,2-PD. AutoDock simulation proposed three potential 1,2-PD binding sites with residue N42, R51, or R146 generating the main interactions (Figure 3A).^26^ For verification, we mutated each of the three residues to Alanine (Ala). As shown in Figure 3B, only R51A completely abolished the responses to 1,2-PD. N42A became less sensitive to 1,2-PD, and R146A exhibited largely reduced activity yet less severe than R51A. Therefore, we speculated that the binding site is most likely located by R51A. Upon further analysis, two more residues proximal to R51, namely, D64 and Q107, also demonstrated potential interactions with 1,2-PD. Mutating D64 to Ala resulted in a disruption of responsiveness to 1,2-PD similar to that seen with R51A, while Q107A showed a diminished sensitivity. These simulations and experimental mutagenesis results strongly supported the most crucial roles of R51 and D64 in binding with 1,2-PD, with Q107 actively contributing as an auxiliary residue. N42 and R146 appeared to be located in the 1,2-PD entry path, and mutating them therefore also affected the responsiveness of PocR to the inducer.

The DBD of PocR engages in more intricate interactions between large molecules, including its binding to the specific DNA sequence and the RNAP. Due to the unique binding box pattern, dissecting the interaction between PocR and its corresponding DNA can be extremely challenging without a crystallized protein structure. On the other hand, we speculated that the interaction between PocR and RNAP may resemble that of other RNAP recruiters. Among the RNAP recruiters with crystallized complex structures, MarA (PDB: 1XS9) exhibits some structural and sequence similarities with PocR^27^ (Figure S2). Therefore, we superimposed the DBD of PocR onto dissected DNA:MarA:RNAP complex (Figure 3C). In MarA, W19 was identified as the most crucial residue for interacting with RNAP, with R36 also playing an active role, likely by forming bonds with N264 and R265 of the α-subunit of RNAP.^27^ The equivalent residues in PocR are Y200 and H217, which possess side chains similar to those of W19 and R36, respectively. Mutating Y200 to Ala led to a complete loss of function, suggesting a detrimental impairment of the interaction with RNAP. H217A is less lethal, with a minor deficiency only present at the lowest IPTG level (Figure 3D). The mutagenesis results are highly analogous to those observed in MarA, including the severe activity inhibition by W19A and the less significant impairment by R36A, which firmly supports the similarity of their RNA polymerase interaction mechanisms.^27^ Therefore, the overlaid structure can serve as valuable guidance for subsequent protein engineering.

We also assessed the performance of the R51A and D64A variants at various expression levels (Figure 3D). Although the inducer binding capabilities of the two variants were completely disrupted, their retention of RNAP-binding ability and the leaky binding with the corresponding promoter resulted in observable activation when expressed at high levels. These findings highlight the distinct roles of these critical residues in PocR, with R51 and D64 responsible for inducer binding and Y199 for RNAP interaction.

### 2.3 Altering the Inducer Specificity of PocR

To further verify the accuracy of the predicted inducer mechanism and enrich the biosensor toolbox, we aimed to engineer the proposed inducer binding pocket of PocR to create a glycerol-inducible biosensor.

**Figure 4:**
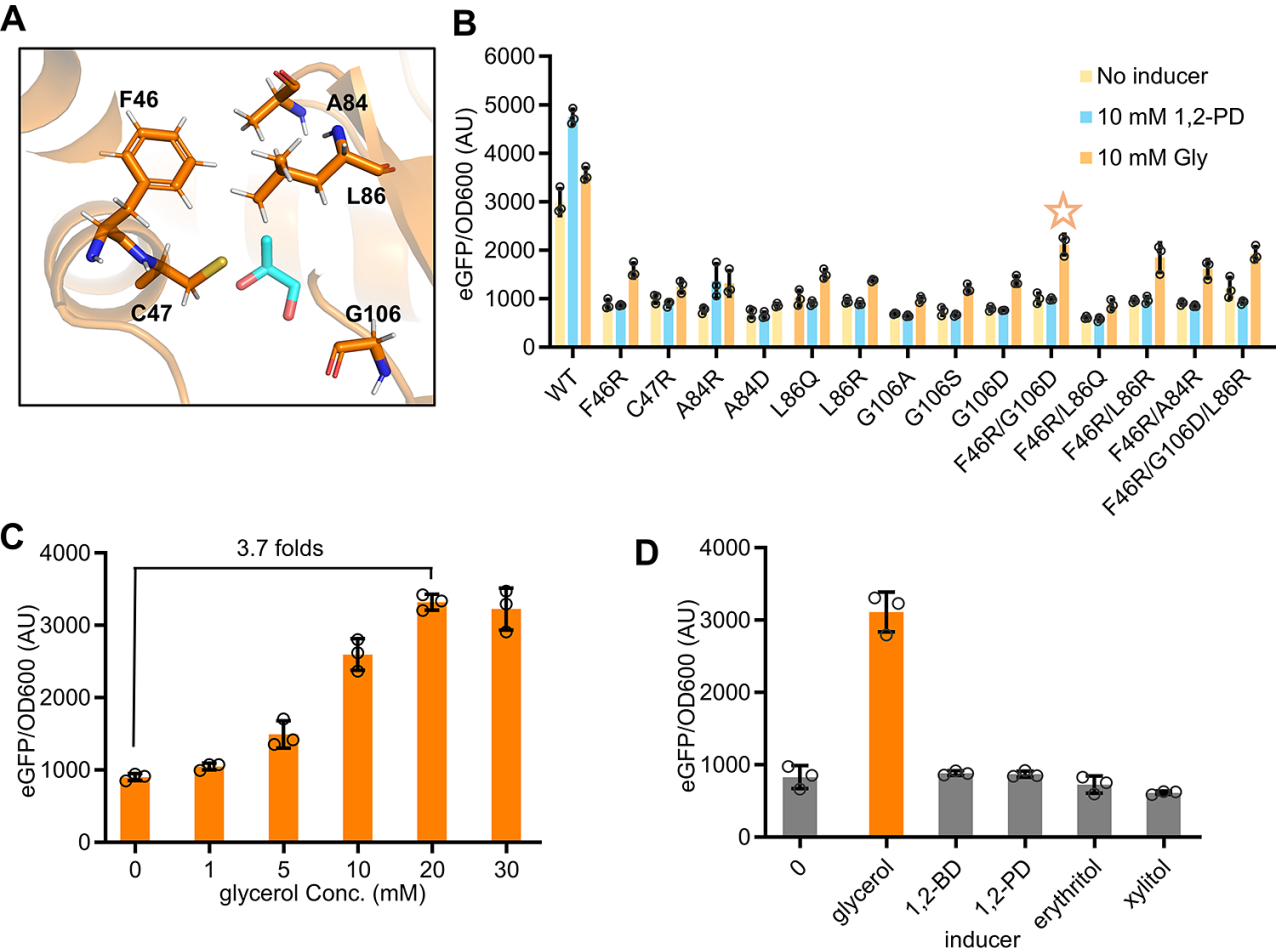
Alteration of the inducer preference. (A) Schematic representation of mutating targets for inducer alteration. (B) Performance of the engineered variants for inducer alteration. (C) Characterization of variant F46R/G106D on increasing concentration of glycerol. (D) Specificity determination of F46R/G106D. All the inducers were added with a concentration of 20 mM. PocR was consistently expressed with 2.5 μM IPTG and all the tests were performed with three independent biological repeats. Individual data points are represented by circles (○), and error bars indicate SD.

Given that 1,2-PD and glycerol share a three-carbon backbone, with the only distinction being the extra hydroxy group in glycerol, we targeted the residues near the methyl group of 1,2-PD within the predicted inducer binding pocket. We hypothesized that improving the hydrophilicity of these residues might enhance the preference for the extra hydroxy group of glycerol (Figure 4A). As shown in Figure 4B, all designed variants exhibited reduced baselines, possibly due to inherent conformational changes in the binding pocket. More importantly, most variants led to higher activation folds triggered by glycerol than the wild type, as expected, and they no longer responded to 1,2-PD. Among all the variants, F46R displayed the highest activation of 1.8-fold with the induction of 10 mM glycerol. Comparatively, wild type PocR (WT) achieved a 1.2-fold activation only under the same glycerol concentration. Furthermore, we combined F46R with other beneficial mutations including G106D, L86Q, L86R, and A84D. The combination of F46R and G106D reached the highest glycerol-induced activation of 2.1-fold, and additionally introducing L86R did not lead to further optimization. Upon more thorough assessment, the PocR-F46R/G106D variant could detect glycerol concentrations ranging from 0 to 20 mM, and correspondingly activate downstream eGFP expression from 901 to 3321 AU, representing a 3.7-fold activation (Figure 4C). Moreover, the variant was confirmed to demonstrate a high specificity to glycerol and could no longer respond to 1,2-PD, 1,2-BD, or other sugar alcohols with similar structures, including erythritol and xylitol (Figure 4D).

Taken together, the predicted inducer binding pocket of PocR and underlying mechanism successfully guided the alteration of its inducer specificity, yielding a glycerol-responsive biosensor with a nearly 4-fold activation dynamic range upon adding 0–20 mM glycerol.

### 2.4 Optimizing the PocR Activation System

**Figure 5:**
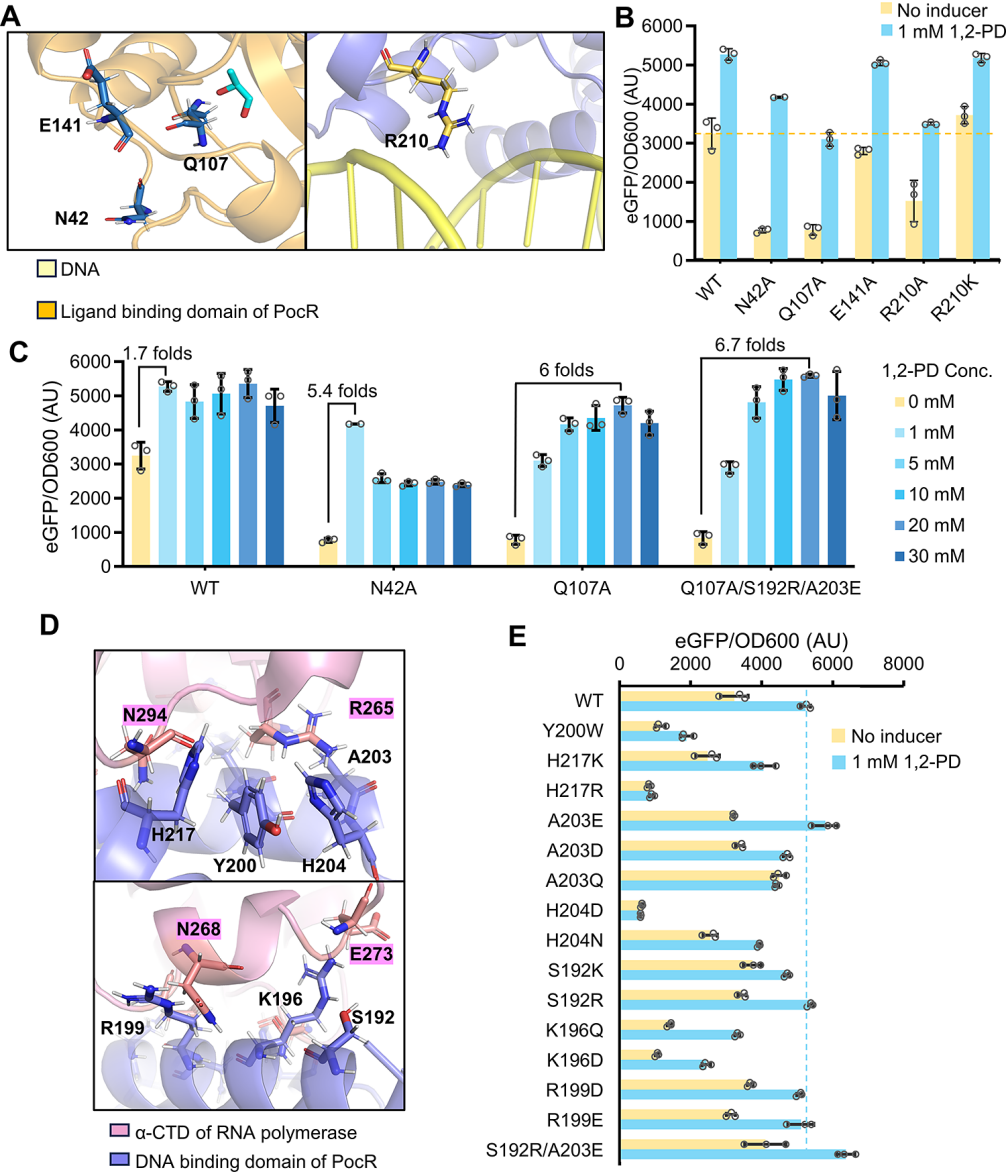
Optimization of the operational range and dynamic range of PocR. (A) Selected mutation targets in PocR that are proximal to the inducer binding pocket or corresponding DNA, respectively. (B) Performance of the engineered variants for less leaky expression. (C) Comprehensive characterization of the optimal variants. (D) Selected engineering targets for enhancing RNAP recruitment. (E) Performance of the engineered variants for stronger interaction with RNAP. PocR was consistently expressed with 2.5 μM IPTG and all the tests were performed with three independent biological repeats. Individual data points are represented by circles (○), and error bars indicate SD.

The highly limited dynamic and operational range of PocR require significant optimization. Leveraging the investigated protein structure and mechanism, we initially targeted the issue of high leaky expression. Our efforts above uncovered that mutating the auxiliary residues within or close to the inducer binding pocket promisingly lowered the leakage baseline, such as Q107 and N42 (Figure 3B). Inspired by this discovery, we re-evaluated the N42A and Q107A constructs and designed another mutation E141A that is likely situated along the 1,2-PD entry path (Figure 5A). Additionally, we explored the possibility of weakening the interaction between PocR and its interactive DNA to reduce leakage further. Despite the lack of an accurate simulation of the specific DNA-PocR interaction, we identified a residue R210 that can potentially form bonds with the DNA backbone nonspecifically. We speculated that mutating this residue might reduce the binding affinity between PocR and its corresponding DNA. Results revealed that N42A and Q107A most effectively decreased the leaky expression from 3250 to 769 and 783 AU and exhibited increased 5.4- and 4.0-fold activation with the addition of 1 mM 1,2-PD, respectively (Figure 5B). E141A remained almost unchanged compared with WT, suggesting an insignificant role. R210 K also showed minimal change, but R210A lowered the leaky expression to 1523 AU and demonstrated a 2.3-fold activation.

The best variants, N42A and Q107A, were characterized using 0–30 mM 1,2-PD to comprehensively measure their operational and dynamic range (Figure 5C). Compared to the WT, N42A exhibited a similar operational range of 0–1 mM 1,2-PD but an improved corresponding dynamic range of 5.4-fold (769–4177 AU). Although showing a lower activated fluorescence level with 1 mM 1,2-PD than N42A, Q107A obtained an expanded operational range of 0–20 mM 1,2-PD, on which its dynamic range increased to 6.0 folds (783–4728 AU).

We noticed that the variants with successfully reduced leaky expression commonly demonstrated lower activated expression levels than the wild type (Figure 5B,C). Therefore, we sought to engineer PocR for a stronger interaction with RNAP to increase the activation efficiency. Specifically, to enhance the interaction with the responsible residue N294 and R265 in RNAP, the two verified residues, Y200 and H217, as well as two surrounding residues, A203 and H204, were first selected for mutating (Figure 5D). In addition, we considered other residues in PocR located on the interface, including S192, K196 and R199, which show promises in interacting with N268 or E273 of RNAP. Across all these mutations, A203E most efficiently improved the 1,2-PD-activated fluorescence expression level, with a 10% increase from 5274 (by WT) to 5883 AU (Figure 5E). The improvement is likely due to the successful generation of new bonds between A203E and residue R265. Mutating S192 to Arginine slightly increased the activated fluorescence from 5274 to 5407 AU, which might be attributed to the new contacts with E273. On the contrary, Y200 appeared to be highly constrained in PocR and even mutating it to a similar residue Tryptophan could cause severe activity loss. Mutations on H217, H204, and K196 might have weakened their original functions and only led to activity loss. R199 is likely nonfunctional, and both the attempted mutations cause almost no activity change. The combination of A203E and S192R synergistically improved the activated fluorescence to 6814 AU, which is 22% higher than that of the wild type. Introducing S192R/A203E to Q107A further improved its dynamic range to 6.7-fold (839–5596 AU), as responses to the operational range of 1–20 mM 1,2-PD. The final mutant PocR-Q107A/S192R/A203E (ARE) thus achieved a nearly 4-fold higher dynamic range and 20-fold wider operational range than the wild type.

### 2.5 Developing a Bifunctional Regulation Circuit Based on PocR

**Figure 6:**
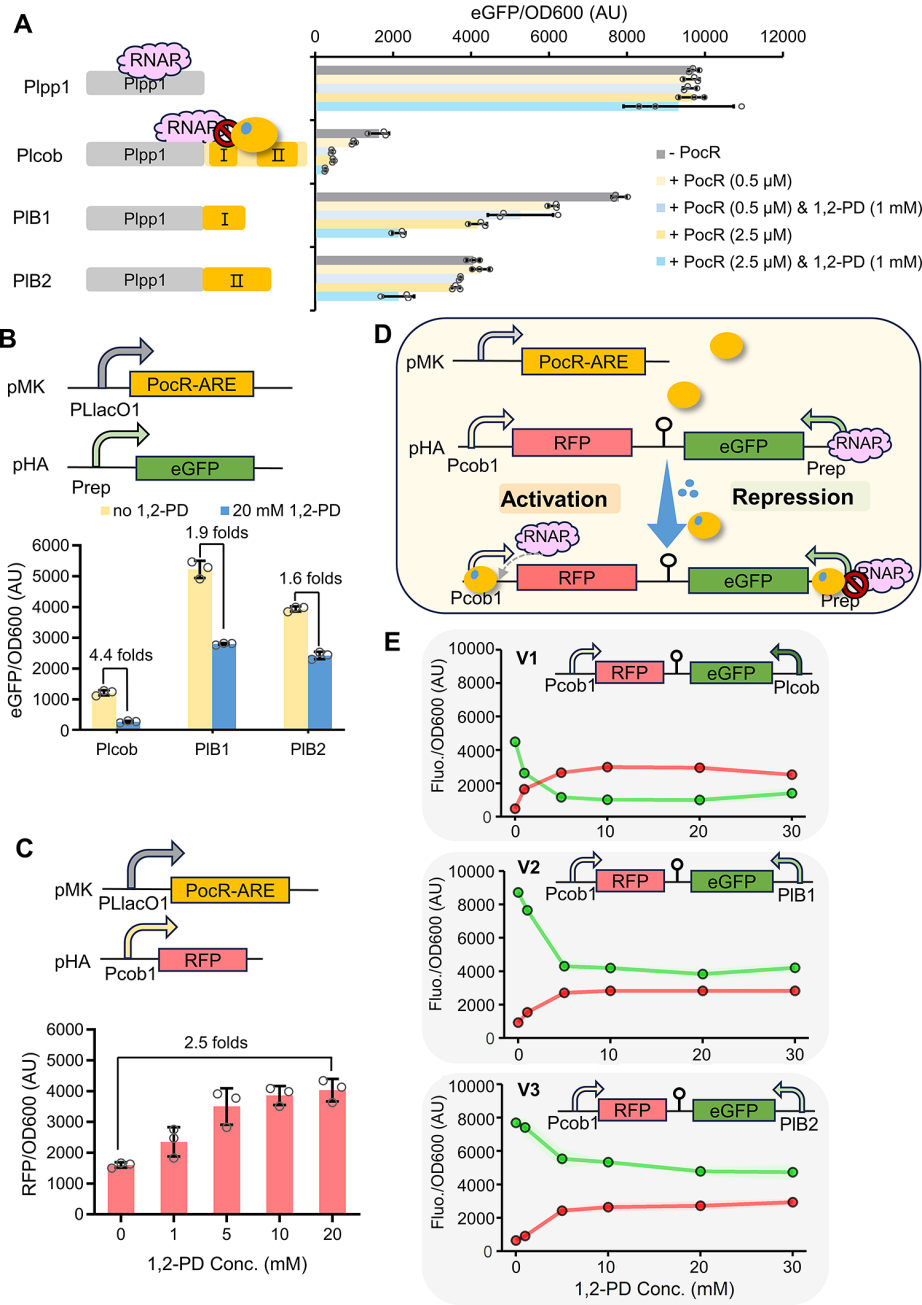
Development of a bifunctional regulation system. (A) Conversion of PocR to a repressor via promoter engineering. (B) Repression is enabled by the optimized variant PocR-ARE. (C) Activation by PocR-ARE using an RFP reporter. In (A–C), individual data points are represented by circles (○), and error bars indicate SD (D) Schematic of the bifunctional regulation system. (E) Performance of the three bifunctional circuits. The shaded regions represent the SD. PocR was consistently expressed with 2.5 μM IPTG unless specified otherwise. All the tests were performed with three independent biological repeats.

Based on the operational mechanism of PocR, we hypothesize that it is possible to broaden the functionality of PocR for gene repression through promoter engineering. By attaching the interactive DNA sequences of PocR to a constitutive promoter, such as Plpp1,^28^ the binding between PocR and the interactive DNA would hinder the movement of RNAP from the upstream Plpp1 promoter, leading to gene repression rather than activation (Figure 6A). To test this hypothesis, we designed the promoter Plcob, PlB1, and PlB2 by linking the Plpp1 promoter sequence to the interactive sequence from box I to box II, single box I, and single box II in Pcob, respectively. As expected, PocR was successfully converted to a repressor on these artificially designed promoters. As a control group, the expression from the Plpp1 promoter remained unaffected by PocR or 1,2-PD, consistently maintaining nearly 10,000 AU (Figure 6A). The expression from Plcob was clearly repressed by the presence of PocR and the further addition of 1,2-PD, though the inserted sequence substantially reduced the expression baseline of Plcob to 1653 AU. PocR expressed by 0.5 μM IPTG repressed the fluorescence level from 1653 to 1002 AU (40% decrease), and the subsequent addition of 1 mM 1,2-PD further reduced the fluorescence to 440 AU. Improving the PocR expression with 2.5 μM IPTG resulted in an over 70% repression to 478 AU compared to the baseline, and continued addition of 1 mM 1,2-PD led to further repression to 269 AU. PlB1 and PlB2 exhibited baselines at 7823 and 4067 AU, respectively, which were closer to that original Plpp1 promoter for the shorter length of inserted sequences. Compared to Plcob, PlB1 and PlB2 showed comparable repression tendencies yet lower repression efficiencies, suggesting weaker binding affinities generated by the single binding box in PlB1 and PlB2. We also identified the potential binding boxes in another PocR-corresponding promoter Ppdu and created PlD1 and PlD2 by connecting Plpp1 to the potential boxes I and II of Ppdu, which demonstrated highly similar performances to PlB1 and PlpB2, respectively (Figure S3).

Next, we assessed the performance of the engineered variant PocR-ARE on the repression promoters Plcob, PlB1, and PlB2 (Figure 6B). The most efficient repression was achieved on Plcob, with a decrease of over 4.4-fold from 1213 to 273 AU. Like the characterization results in Figure 6A, PlB1 and PlB2 exhibited higher baseline fluorescence levels at 5224 and 3937 AU before repression, yet the repression efficiencies were lower as 1.9-fold and 1.6-fold, respectively.

Encouraged by the established repression system, we continued to explore the feasibility of developing a bifunctional regulation system using PocR. To achieve this, we incorporated RFP (red fluorescence) as the reporter for the activation module. Upon the addition of 0–20 mM 1,2-PD, PocR-ARE activated RFP expression from 1599 to 4032 AU (Figure 6C). This 2.5-fold activation is lower than the dynamic range obtained using eGFP, possibly due to differences in translational processing between the two reporter genes. The activation module was integrated with the three repression modules, respectively, generating three versions of bifunctional regulation circuits named V1, V2, and V3 (Figure 6D). Upon testing, all designs achieved 1,2-PD triggered bifunctional regulation, confirming the compatibility between the activation and the repression modules. The three versions of circuits led to versatile performances (Figure 6E). Specifically, upon addition of 0–20 mM 1,2-PD, V1 showed RFP activation from 482 to 2932 AU (6-fold) and eGFP repression from 4492 to 1007 AU (4.5-fold). The activation and repression fold changes were surprisingly more pronounced than those observed in single regulation, probably due to the division of PocR-ARE on the two corresponding promoters, which further lowers the leaky binding (Figure 6B,C,E). Similar phenomena were presented on circuits V2 and V3 likely for the same reason. V2 showed a 3.4-fold activation ranging from 820 to 2827 AU and a 2.3-fold repression from 8728 to 3837 AU in response to 0–20 mM 1,2-PD (Figures 6E and S4). Likewise, V3 demonstrated a 4.2-fold activation from 646 to 2727 AU and a 1.6-fold repression from 7694 to 4780 AU over the same operational range. Overall, these results showcased the successful development of a versatile bifunctional regulation system based on a single PocR regulator and significantly enhanced the applicability of PocR.

## 3 Discussion

This study investigated and engineered a 1,2-PD-responsive biosensor PocR to address the limitations of the TF-based biosensor repertoire in short-chain alcohols. Comprehensive *in vivo* characterization uncovered the highly limited dynamic range and operational range of PocR, which impedes further practical application. Structure and mechanism investigations supported the rational engineering for PocR optimization, generating an ARE variant with a 20-fold broader operational range and a 4-fold higher dynamic range. In addition, promoter engineering enabled the successful conversion of PocR from a transcription activator to a repressor. Integration of the repression and activation modules successfully developed a versatile 1,2-PD-induced bifunctional regulation system relying on a single regulator PocR-ARE. These engineered variants and regulation systems hold significant potentials for metabolic engineering applications such as dynamic regulation and high-throughput screening.

Previous studies have identified PocR as an AraC family transcription factor capable of responding to 1,2-PD and activating the expression of the Cob operon for vitamin B biosynthesis and the Pdu operon for propanediol degradation.^22−24^ Using the PocR regulation system established in *E. coli*, we observed its responsiveness not only to 1,2-PD but also to glycerol and 1,2-butanediol (1,2-BD), which explains the glycerol induction of the Cob and Pdu operons previously observed in the original host *S. typhimurium*.^23^ Rational engineering created a PocR-F46R/G106D variant specifically responsive to glycerol, suggesting the plasticity of the ligand-binding pocket and the potential of PocR to be engineered to sense other short-chain alcohols or chemicals with similar structures.

While the dissected RNAP recruitment mechanism of MarA significantly facilitated our investigation into PocR, the activation mechanism of PocR has yet to be completely uncovered, such as the conformational changes caused by the binding with 1,2-PD that could lead to stronger activation. Moreover, the interaction between PocR and its corresponding promoters remains unclear. The two binding boxes in Pcob have been confirmed *in vitro*, which are 20 bp and 48 bp in length with a distance of 52 bp.^22^ We also identified two potential binding boxes in the Ppdu promoter during exploration, with respective sequence similarities of 80% and 56% to those in Pcob but separated by a longer distance of 215 bp (Figure S3). Such operational DNA pattern is significantly distinctive from the operational DNA of other reported transcription activators in the AraC family.^27,29−34^ Moreover, our attempt to enhance the activation activity through promoter hybridization yielded only a barely functional promoter, indicating the complexity of the activation mechanism of PocR (Figure S5). Therefore, the crystallization of the PocR functional complexes, particularly those involving PocR and its corresponding DNA, is highly demanded to acquire a concrete understanding of this activator and support more diverse relevant exploitations in the future.

Many biosensor-enabled inducible bifunctional regulation circuits have been developed, offering versatile performances to meet various regulatory needs in applications.^35−40^ However, these bifunctional circuits were typically established by harnessing two orthogonal regulation systems or by combining a biosensor with a dCas9-based regulator. In comparison, the PocR biosensor with RNAP recruiting capability demonstrated unique advantages in function expansion. Through simple promoter redesign, it can be easily transformed into a repressor or bifunctional regulator, providing more versatile functions and performances. We anticipate more exploration and exploitation of inducible RNAP recruiters such as PocR to enrich the biosensor toolbox.

## 4 Method and Material

### 4.1 Strain, Plasmids, and Chemicals

All of the strains and plasmids used in this study were listed in Supporting Information Table 1. The *E. coli* strain XL1-blue (Stratagene, La Jolla, CA) was used for plasmid construction and extraction and BW25113 (F′) was used for *in vivo* PocR performance determination. Plasmid pHA-MCS (high copy number) and pMK-MCS (medium copy number) were used for gene expression.^41^ PocR and its corresponding promoters were obtained from the genomic DNA of *Salmonella typhimurium* LT2 (ATCC 700720D-5).

All of the chemicals were procured from Sigma. Phusion DNA polymerase, as well as all restriction endonucleases, and the Quick Ligation Kit were purchased from New England Biolabs (Beverly, MA, USA). Additionally, the Plasmid Miniprep Kit, Gel Recovery Kit, and DNA Cleanup Kit were sourced from Zymo Research (Irvine, CA, USA).

### 4.2 Plasmid Construction

PocR was inserted into the medium-copy pMK-MCS plasmid between *Kpn*I and *Bam*HI sites, resulting in the creation of pMK-pLlacO1-PocR. The pMK plasmids containing PocR variants were constructed using the SLIM method and confirmed by Sanger sequencing.^42^

Reporter plasmids were generated by inserting a Pcob or Ppdu promoter into the high-copy pHA-eGFP-MCS plasmid using *XhoI* and *Eco*RI restriction enzymes to replace the PLlacO1 promoter, resulting in pHA-Pcob-eGFP and pHA-Ppdu-eGFP, respectively. Truncated or hybridized promoters were obtained by PCR amplification of pHA-Pcob-eGFP and pHA-Ppdu-eGFP, followed by similar digestion with *XhoI* and *Eco*RI, and subsequent cloning into the pHA-eGFP-MCS plasmid. The plasmid pHA-Pcob1-RFP was constructed by replacing eGFP in pHA-Pcob1-eGFP with *Kpn*I and *Sal*I digested RFP. The complete operons of Prep-eGFP-T1 (Prep refers to Plcob, PlB1 or PlB2) were amplified by PCR, digested with *SpeI* and *XhoI*, and then inserted into pHA-Pcob1-RFP between the NheI and *Sal*I sites, resulting in the creation of pHA-Pcob1-RFP-Plcob-eGFP (V1), pHA-Pcob1-RFP-PLB1-eGFP (V2), and pHA-Pcob1-RFP-PLB2-eGFP (V3).

### 4.3 Stain Cultivation

*E. coli* strains were cultivated at 37 °C in Luria–Bertani (LB) medium containing 5 g/L yeast extract, 10 g/L NaCl, and 10 g/L tryptone, supplemented with the appropriate antibiotics as needed. For the PocR performance test, 3 mL of LB medium was cultured in a rotary shaker at 270 rpm for 24 h. Ampicillin (Amp) and/or kanamycin (Kan) antibiotics were added to the medium at final concentrations of 100 and 50 μg/mL, respectively. Isopropyl β-D-1-thiogalactopyranoside (IPTG) and 1,2-PD, or other inducers, were added at the concentrations described above.

### 4.4 PocR Performance Determination

To assess the performance of PocR or its derivative variants on their original or engineered corresponding promoters, pMK plasmids containing PocR or its variants were cotransformed with the reporter plasmids into *E. coli* BW25113 (F′). The empty plasmid pMK-MCS served as the negative control without PocR when necessary. Transformants were plated on LB agar plates supplemented with Amp and Kan antibiotics and incubated overnight. Single colonies were selected and inoculated into 3 mL LB medium containing appropriate antibiotics, IPTG, and the inducer (1,2-PD unless otherwise specified). After 24 h, 20 μL of culture was sampled and mixed with 180 μL distilled water in a black 96-well plate for cell density and fluorescence detection using a Synergy microplate reader (BioTek, Winooski, VT). Cell density was measured at a wavelength of 600 nm (OD600). Green fluorescence (eGFP) was detected with an excitation wavelength of 485 nm and an emission wavelength of 528 nm, while red fluorescence with an excitation wavelength of 530 nm and an emission wavelength of 590 nm. The eGFP/OD600 or RFP/OD600 ratio was calculated as (fluorescence – background)/[(OD600 – background) × 1.76].^41,43,44^ All of the tests were performed using three biological repeats.

## Supporting Information Available

Strains and plasmids used in this study (Table S1); 1,2-PD induction characterization of Ppdu promoter (Figure S1); sequence alignment between the DNA binding domain of PocR and MarA (Figure S2); repression promoter comparison (Figure S3); bifunctional regulation by V2 circuit (Figure S4); characterization of PBlpp0.03 promoter (Figure S5) (PDF)
