# Supplementary material for: Investigating and Engineering an 1,2-Propanediol-Responsive Transcription Factor-Based Biosensor
Source: ACS Synth Biol. 2024 Jul 5;13(7):2177–87. doi: 10.1021/acssynbio.4c00237 (PMC11264322; doi:10.1021/acssynbio.4c00237)
Supplement: Supplementary file 1 — sb4c00237_si_001.pdf [file sb4c00237_si_001.pdf]

Supporting Information

Investigating and engineering an 1,2-propanediol-responsive transcription factor-based biosensor

Yuxi Teng[a], Xinyu Gong[a], Jianli Zhang[a], Ziad Obideen[b], Yajun Yan[a, 1]

[a] School of Chemical, Materials and Biomedical Engineering, College of Engineering, The University of Georgia, Athens, GA 30602, USA.

[b] Franklin College of Arts and Sciences, The University of Georgia, Athens, GA 30602, USA.

[1] Corresponding author: Yajun Yan

Address: 2040E Interdisciplinary STEM Research Building 1, 302 East Campus Road, Athens, GA 30602, USA

E-mail: yajunyan@uga.edu; telephone: +1-706-542-8293

Table S1. Strains and plasmids used in this study.

Strains	Description	Reference
E. coli XL-1 Blue	*recA*1 *endA*1 *gyrA*96 *thi*-1 *hsdR*17 *supE*44 *relA*1 *lac F'* [*traD36 proAB lacI*^{q}*Z*Δ*M*15 *Tn*10 (*Tet*^{r})]	Stratagene
E. coli BW25113 (F')	*rrnBT*14 Δ*lacZWJ*16 *hsdR*514 *ΔaraBADAH*33 *ΔrhaBADLD*78 *F'* [*traD36 proAB lacI*^{q}*ZΔM15 Tn10(Tet*^{r}*)*]	[1]
**Plasmids**	**Description**	**Reference**
pHA-MCS	P_L*lacO1*, multiple cloning sites, *ColE1 ori*, *Amp*^{R}	[2]
pHA-eGFP-MCS	P_L*lacO1*, eGFP, multiple cloning sites, *ColE1 ori*, *Amp*^{R}	[3]
pMK-MCS	P_L*lacO1*, multiple cloning sites, *p15Aori*, *Kan*^{R}	[4]
pMK-PLlacO1-PocR	P_L*lacO1*, *PocR*, *p15Aori*, *Kan*^{R}	This study
pMK-PLlacO1-PocR-N42A	pMK-PLlacO1-PocR plasmid with PocR harboring N42A mutation	This study
pMK-PLlacO1-PocR-R146A	pMK-PLlacO1-PocR plasmid with PocR harboring R146A mutation	This study
pMK-PLlacO1-PocR-R51A	pMK-PLlacO1-PocR plasmid with PocR harboring R51A mutation	This study
pMK-PLlacO1-PocR-D64A	pMK-PLlacO1-PocR plasmid with PocR harboring D64A mutation	This study
pMK-PLlacO1-PocR-Q107A	pMK-PLlacO1-PocR plasmid with PocR harboring Q107A mutation	This study
pMK-PLlacO1-PocR-Y200A	pMK-PLlacO1-PocR plasmid with PocR harboring Y200A mutation	This study
pMK-PLlacO1-PocR-H217A	pMK-PLlacO1-PocR plasmid with PocR harboring H217A mutation	This study
pMK-PLlacO1-PocR-F46R	pMK-PLlacO1-PocR plasmid with PocR harboring F46R mutation	This study
pMK-PLlacO1-PocR-C47R	pMK-PLlacO1-PocR plasmid with PocR harboring C47R mutation	This study
pMK-PLlacO1-PocR-A84R	pMK-PLlacO1-PocR plasmid with PocR harboring A84R mutation	This study
pMK-PLlacO1-PocR-A84D	pMK-PLlacO1-PocR plasmid with PocR harboring A84D mutation	This study
pMK-PLlacO1-PocR-L86Q	pMK-PLlacO1-PocR plasmid with PocR harboring L86Q mutation	This study

pMK-PLlacO1-PocR-L86R	pMK-PLlacO1-PocR plasmid with PocR harboring L86R mutation	This study
pMK-PLlacO1-PocR-G106A	pMK-PLlacO1-PocR plasmid with PocR harboring G106A mutation	This study
pMK-PLlacO1-PocR-G106S	pMK-PLlacO1-PocR plasmid with PocR harboring G106S mutation	This study
pMK-PLlacO1-PocR-G106D	pMK-PLlacO1-PocR plasmid with PocR harboring G106D mutation	This study
pMK-PLlacO1-PocR-F46R/G106D	pMK-PLlacO1-PocR plasmid with PocR harboring F46R and G106D mutations	This study
pMK-PLlacO1-PocR-F46R/L86Q	pMK-PLlacO1-PocR plasmid with PocR harboring F46R and L86Q mutations	This study
pMK-PLlacO1-PocR-F46R/L86R	pMK-PLlacO1-PocR plasmid with PocR harboring F46R and L86R mutations	This study
pMK-PLlacO1-PocR-F46R/A84R	pMK-PLlacO1-PocR plasmid with PocR harboring F46R and A84R mutations	This study
pMK-PLlacO1-PocR-F46R/G106D/L86R	pMK-PLlacO1-PocR plasmid with PocR harboring F46R, L106D, and L86R mutations	This study
pMK-PLlacO1-PocR-R210A	pMK-PLlacO1-PocR plasmid with PocR harboring R210A mutation	This study
pMK-PLlacO1-PocR-R210K	pMK-PLlacO1-PocR plasmid with PocR harboring R210K mutation	This study
pMK-PLlacO1-PocR-Y200W	pMK-PLlacO1-PocR plasmid with PocR harboring Y200W mutation	This study
pMK-PLlacO1-PocR-H217K	pMK-PLlacO1-PocR plasmid with PocR harboring H217K mutation	This study
pMK-PLlacO1-PocR-H217R	pMK-PLlacO1-PocR plasmid with PocR harboring H217R mutation	This study
pMK-PLlacO1-PocR-A203E	pMK-PLlacO1-PocR plasmid with PocR harboring A203E mutation	This study
pMK-PLlacO1-PocR-A203D	pMK-PLlacO1-PocR plasmid with PocR harboring A203D mutation	This study
pMK-PLlacO1-PocR-A203Q	pMK-PLlacO1-PocR plasmid with PocR harboring A203Q mutation	This study
pMK-PLlacO1-PocR-H204D	pMK-PLlacO1-PocR plasmid with PocR harboring H204D mutation	This study
pMK-PLlacO1-PocR-H204N	pMK-PLlacO1-PocR plasmid with PocR harboring H204N mutation	This study
pMK-PLlacO1-PocR-S192K	pMK-PLlacO1-PocR plasmid with PocR harboring S192K mutation	This study

pMK-PLlacO1-PocR-S192R	pMK-PLlacO1-PocR plasmid with PocR harboring S192R mutation	This study
pMK-PLlacO1-PocR-K196Q	pMK-PLlacO1-PocR plasmid with PocR harboring K196Q mutation	This study
pMK-PLlacO1-PocR-R199D	pMK-PLlacO1-PocR plasmid with PocR harboring R199D mutation	This study
pMK-PLlacO1-PocR-R199E	pMK-PLlacO1-PocR plasmid with PocR harboring R199E mutation	This study
pMK-PLlacO1-PocR-S192R/A203E	pMK-PLlacO1-PocR plasmid with PocR harboring S192R and A203E mutation	This study
pMK-PLlacO1-PocR-Q107A/S192R/A203E	pMK-PLlacO1-PocR plasmid with PocR harboring Q107A, S192R and A203E mutations	This study
pHA-Pcob-eGFP	pHA with Pcob controlled eGFP	This study
pHA-Ppdu-eGFP	pHA with Ppdu controlled eGFP	This study
pHA-Pcob1-eGFP	pHA with pcob1 controlled eGFP. Pcob1 was generated by eliminating the sequence downstream the transcription starting stie in Pcob by a reverser primer 5'-ggaagaattCTGACACTGTACTTCATCACA-3'	This study
pHA-Pcob2-eGFP	pHA with Pcob2 controlled eGFP. Pcob2 was generated by removing the binding box I in Pcob1 promoter by a forward primer 5'-ggaactcgagAAAATTTATCTGGTGTAACAA-3'	This study
pHA-Pcob3-eGFP	pHA with Pcob3controlled eGFP. Pcob3 was generated by replacing the binding box II in Pcob1 promoter by a random sequence TATACGACCATACCATTCGAGCATGGCACTATGTACGCTGTCCCCATT	This study
pHA-Plpp1-eGFP	pHA with Plpp1 controlled eGFP	This study
pHA-Plcob-eGFP	pHA with PLcob controlled eGFP	This study
pHA-PlB1-eGFP	pHA with PLB1 controlled eGFP	This study
pHA-PlB2-eGFP	pHA with PLB2 controlled eGFP	This study
pHA-PlD1-eGFP	pHA with PLD1 controlled eGFP	This study
pHA-PlD2-eGFP	pHA with PLD2 controlled eGFP	This study
pHA-PlD3-eGFP	pHA with PLD3 controlled eGFP	This study
pHA-Pcob1-RFP	pHA with Pcob1 controlled RFP	This study

pHA-Pcob1-RFP-PLcob-eGFP (V1)	pHA with a Pcob1 controlled RFP and a PLcob controlled eGFP, separate by a shared T1 terminator	This study
pHA-Pcob1-RFP-PLB1-eGFP (V2)	pHA with a Pcob1 controlled RFP and a PLB1 controlled eGFP, separate by a shared T1 terminator	This study
pHA-Pcob1-RFP-PLB2-eGFP (V3)	pHA with a Pcob1 controlled RFP and a PLB2 controlled eGFP, separate by a shared T1 terminator	This study
pHA-PBlpp0.03-eGFP	pHA with PBlpp0.03 promoter controlled eGFP	This study

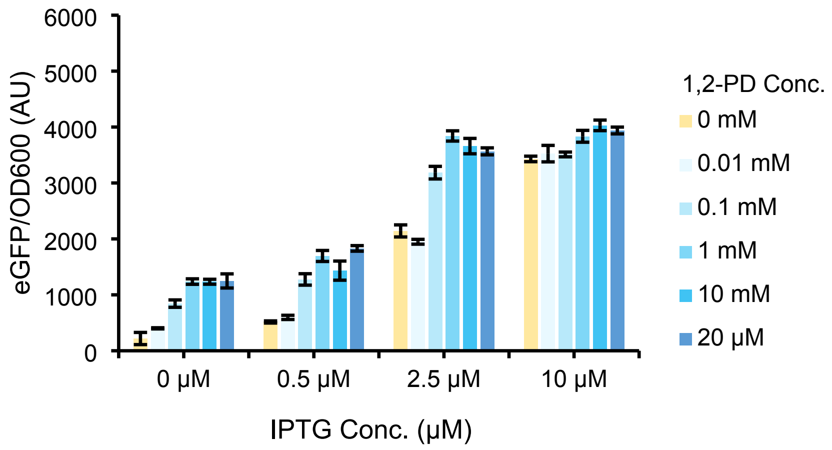

Figure S1. 1,2-PD induction characterization on the Ppdu promoter

```
CLUSTAL O(1.2.4) multiple sequence alignment


PocR      TPHDSKMKKALRYIDAHLSDDLRLEDVASHVYLSPYYFSKLFKKYQGIGFNAWVNRQRMV  60
MarA      NTDAITIHSILDWIEDNLESPLSLEKVSERSGYSKWHLQRMFKKETGHSLGQYIRSRKMT  60
          . .  .::. * :*: :*.. * **.*:.:   * :::.::***  * .:. ::. ::*.

PocR      SARELLCHSDWSIASIARNLGFSQTSYFCKVFRQTYQVTPQAYRQQINENSH----PPSL  116
MarA      EIAQKLKESNEPILYLAERYGFESQQTLTRTFKNYFDVPPHKYRMTNMQGESRFLHPLNH  120
          .  : * .*:  *  :*.. **.. . : :.*:: ::* *: **    :..     * .

PocR      ---   116
MarA      YNS   123
```

Figure S2. Sequence alignment between the DNA binding domain of PocR and MarA. * indicates completely identical residue; : indicates strongly similar properties of the aligned residues; . indicates weakly similar properties of the aligned residues.

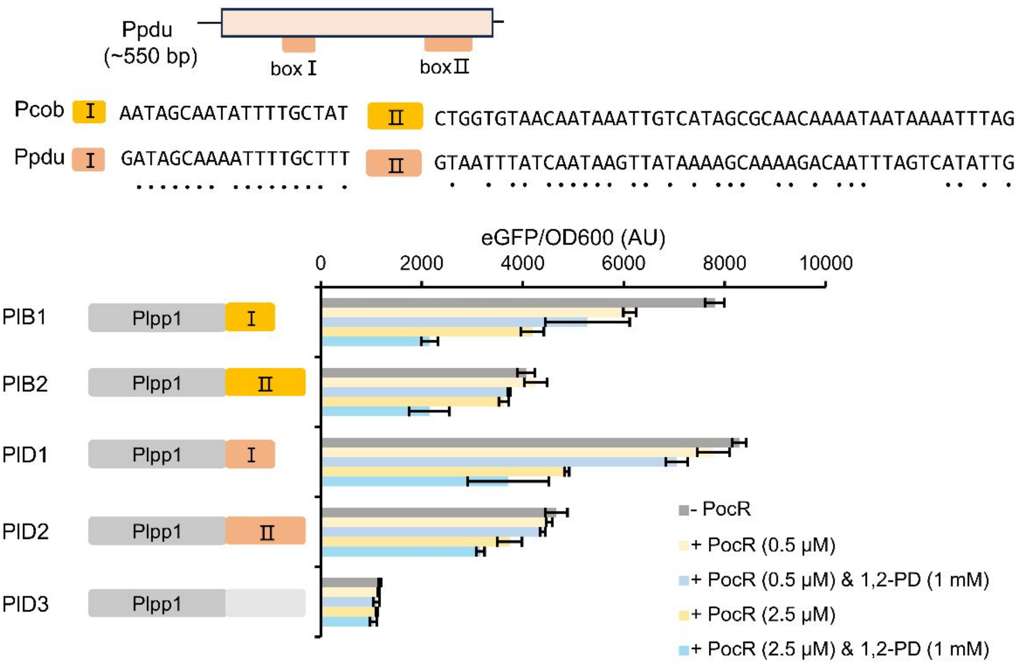

Figure S3. Comparison between PIB1, PIB2 with PID1 and PID2. PID3 contains another potential binding box II in Ppdu showing sequence similarity with Pcob-Box II, which was found to be non-functional.

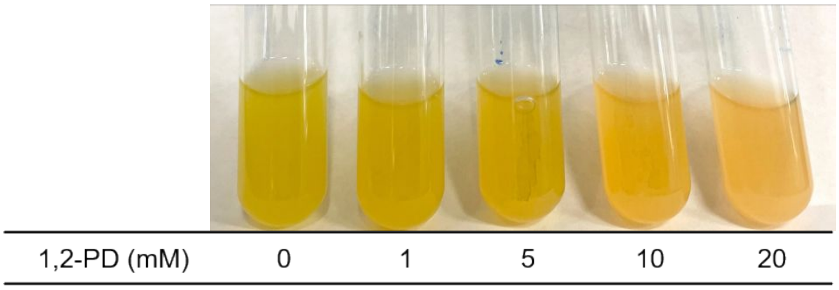

Figure S4. Bifunctional regulation demonstrated by PocR on the V2 circuit

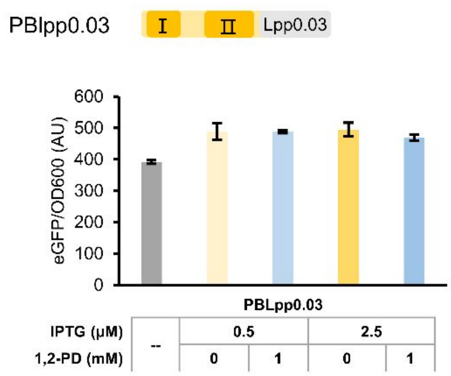

Figure S5. Characterization of PBlpp0.03 promoter

Reference

1. Atsumi, S.; Cann, A. F.; Connor, M. R.; Shen, C. R.; Smith, K. M.; Brynildsen, M. P.; Chou, K. J.; Hanai, T.; Liao, J. C., Metabolic engineering of Escherichia coli for 1-butanol production. *Metabolic engineering* **2008,** *10* (6), 305-311.
2. Jiang, T.; Li, C.; Zou, Y.; Zhang, J.; Gan, Q.; Yan, Y., Establishing an Autonomous Cascaded Artificial Dynamic (AutoCAD) regulation system for improved pathway performance. *Metab Eng* **2022,** *74*, 1-10.
3. Li, C.; Zhou, Y.; Zou, Y.; Jiang, T.; Gong, X.; Yan, Y., Identifying, Characterizing, and Engineering a Phenolic Acid-Responsive Transcriptional Factor from Bacillus amyloliquefaciens. *ACS Synth Biol* **2023,** *12* (8), 2382-2392.
4. Jiang, T.; Teng, Y.; Li, C.; Gan, Q.; Zhang, J.; Zou, Y.; Desai, B. K.; Yan, Y., Establishing Tunable Genetic Logic Gates with Versatile Dynamic Performance by Varying Regulatory Parameters. *ACS Synthetic Biology* **2023,** *12* (12), 3730-3742.
